# Enhancing Glucuronic Acid and Bacterial Cellulose Yield in Kombucha via Valorization of Male Jelly Fig (*Ficus pumila* L. var. *awkeotsang*)

**DOI:** 10.3390/foods15081290

**Published:** 2026-04-09

**Authors:** Yu-Chieh Chou, Wei-Lun Ku, Kuan-Chen Cheng, Chen-Che Hsieh, Shella Permatasari Santoso, Yung-Kai Lin, Wei-Lun Hung, Shin-Ping Lin

**Affiliations:** 1 Ph.D. Program in Drug Discovery and Development Industry, College of Pharmacy, Taipei Medical University, 250 Wu-Hsing Street, Taipei 11031, Taiwan; 2 School of Food Safety, Taipei Medical University, 250 Wu-Hsing Street, Taipei 11031, Taiwan; 3 Institute of Biotechnology, National Taiwan University, Taipei 10617, Taiwan; 4 Institute of Food Science and Technology, National Taiwan University, Taipei 10617, Taiwan; 5 Department of Optometry, Asia University, Taichung 41354, Taiwan; 6 Department of Food Science, Fu Jen Catholic University, New Taipei City 242062, Taiwan; 7 Department of Medical Research, China Medical University Hospital, China Medical University, Taichung 40402, Taiwan; 8 Department of Seafood Science, College of Hydrosphere, National Kaohsiung University of Science and Technology, Kaohsiung 81157, Taiwan; 9 Department of Chemical Engineering, Faculty of Engineering, Widya Mandala Surabaya Catholic University, Surabaya 60114, Indonesia; 10 Chemical Engineering Master Program, Widya Mandala Surabaya Catholic University, Surabaya 60114, Indonesia; 11 Collaborative Research Center for Zero Waste and Sustainability, Widya Mandala Surabaya Catholic University, Surabaya 60114, Indonesia; 12 Institute of Food Safety and Risk Management, National Taiwan Ocean University, Keelung City 20224, Taiwan; 13 Research Center of Biomedical Device, Taipei Medical University, 250 Wu-Hsing Street, Taipei 11031, Taiwan

**Keywords:** kombucha, male jelly fig, bacterial cellulose

## Abstract

Male jelly fig (*Ficus pumila* L. var. *awkeotsang*) syconia are an underutilized by-product in Taiwan. This study evaluated male fig powder (0–2%, *w*/*v*) as a substrate for producing male fig-altered kombucha (FK) using a defined co-culture of *Komagataeibacter xylinus* and *Saccharomyces cerevisiae*. Fermentation markedly reshaped FK metabolites. Glucuronic acid increased in a dose-associated manner, reaching 6.63 g/L in 2% FK, whereas vitamin C declined during fermentation but remained highest in 2% FK. Gallic acid increased and peaked at 0.5% FK (320.75 mg/L), while acetic and succinic acids showed formulation-dependent patterns; conversely, caffeine decreased in all male fig-containing groups. FK also exhibited concentration-dependent color divergence from the control at day 9 (ΔE* up to 17.81 at 2% FK). Numerical increases in DPPH and TPC were observed; however, no significant differences were detected among the treatments. Importantly, male fig supplementation substantially enhanced kombucha bacterial cellulose (KBC) yield (0.56 to 7.28 g/L from 0 to 2% FK) without compromising high water content (~96–99%) or swelling (~90–94%). FTIR confirmed retention of the cellulose backbone, SEM showed formulation-dependent fibril diameters, and TGA indicated higher early-stage thermal stability with dose-dependent shifts in main degradation behavior. Collectively, male figs can be valorized to produce FK with altered metabolite profiles and improved KBC productivity.

## 1. Introduction

Traditional kombucha is a fermented beverage produced by inoculating sweetened green or black tea with a symbiotic culture of bacteria and yeast (SCOBY) [1]. Beyond its distinctive sensory attributes, kombucha has been investigated for several reported bioactivities, including antioxidant capacity [2], antimicrobial effects [3], anti-inflammatory potential [4], antidiabetic activities [5], anticarcinogenic effects [6], and probiotic potential [7]. Within the SCOBY consortium, yeasts ferment sugars to generate carbon dioxide, ethanol, and other metabolites, contributing to carbonation and aroma complexity [8,9]. Acetic acid bacteria (AAB) (e.g., *Komagataeibacter xylinus*) synthesize a bacterial cellulose (BC) pellicle that is associated with the characteristic mouthfeel of kombucha and represents a valuable biopolymer [10]. In addition, organic acids formed by AAB can modulate the sensory profile through co-culture interactions with yeasts, promoting the formation of characteristic aromatic compounds [11].

Food and agro-industrial wastes are increasingly recognized as major sustainability challenges, as they represent the embedded environmental costs of agricultural production, processing, and distribution, while their subsequent disposal may additionally contribute to greenhouse-gas emissions and leachate-related contamination [12,13]. Consequently, transforming underutilized biomass into value-added products aligns with circular economy strategies, where agricultural by-products (peels, seeds, pulp, and other residues) can serve as functional ingredients or substrates for bioprocessing rather than disposal streams [14,15]. Among the available approaches, fermentation is particularly attractive because it can improve extractability, release bound phytochemicals, and generate new metabolites, thereby “upcycling” by-products into foods and ingredients with enhanced functionality [16].

Jelly fig (*Ficus pumila* L. var. *awkeotsang*) is closely associated with Taiwan and has been described as likely endemic in Taiwan based on host–pollinator biogeography and phylogeographic evidence [17]. The seed-derived polysaccharide fraction is rich in low methoxyl pectin, which can form gels at ambient temperature through ion-mediated crosslinking and enzyme-coupled network formation, explaining the unique gelation behavior of aiyu jelly [18]. *F. pumila* exhibits functional dioecy, in which male syconia primarily support pollinating fig wasps and provide pollen, whereas female syconia yield the seed crop [17]. From a valorization perspective, male syconia constitute a potentially underutilized biomass stream after fulfilling their reproductive function While the achenes of *F. pumila* var. *awkeotsang* are known to be rich in low methoxyl pectin and various bioactive phytochemicals have been reported in different plant tissues, the chemical composition of male syconia has not been well characterized, motivating systematic evaluation [18].

Given the metabolic breadth of SCOBY consortia, kombucha fermentation has been increasingly used as a versatile biotransformation platform for herbal materials and agricultural by-products, enabling the development of novel functional beverages and co-products [19,20,21,22]. Common strategies include incorporating polyphenol-rich fruit matrices to intensify antioxidant potential [23] and fermenting with medicinal herbs to further potentiate inherent bioactivities [24,25]. Notably, modifying substrates can also alter the physicochemical properties of kombucha-derived BC, indicating that fermentation inputs may reshape BC microstructure and performance [16,26].

In the present study, male jelly fig syconia were utilized as a fermentation substrate to develop male fig-altered kombucha (FK). We evaluated fermentative metabolites and antioxidant properties of FK, as well as further investigating the material characteristics of kombucha bacterial cellulose (KBC), including water content, swelling behavior, morphology, and chemical structure. Future work should integrate multi-omics profiling of SCOBY dynamics with targeted metabolomics to clarify which microbial interactions drive bioactive formation, while simultaneously addressing safety, sensory acceptability, and process scale-up to facilitate sustainable, circular utilization of jelly fig by-products [13,16].

## 2. Materials and Methods

### 2.1. Microorganisms and Maintenance

*Komagataeibacter xylinus* (*K. xylinus*) ATCC 700178 and *Saccharomyces cerevisiae* (*S. cerevisiae*) ATCC 21447 were obtained from the Bioresource Collection and Research Center (BCRC; Hsinchu City, Taiwan) and preserved at −80 °C until use. Corn steep liquor (CSL)–fructose medium (Jing-Ming Co., Ltd., Nan Tou City, Taiwan; proprietary formulation provided by the manufacturer) and yeast–peptone–dextrose (YPD) medium (yeast extract 10 g/L, peptone 20 g/L, and dextrose 20 g/L) were sterilized by autoclaving at 121 °C for 40 min. For bacterial activation, *K. xylinus* was inoculated into 50 mL of CSL–fructose medium and incubated statically at 28 °C for 3 days. In parallel, *S. cerevisiae* was inoculated into 50 mL of YPD medium and incubated statically at 28 °C for 2 days.

### 2.2. Preparation and Fermentation of FK

Male figs used in this study were obtained from Hongjiu Ai-yu Garden (Pingtung City). The male figs were cut into ~2 cm^3^ pieces, freeze-dried, milled, and passed through a sieve to obtain a homogeneous powder, which was stored at −20 °C until use. For fermentation, a black tea infusion (1% *w*/*v*) was prepared by boiling 1 L of distilled water and steeping black tea leaves. The resulting tea was formulated with male fig powder at 0–2% (*w*/*v*) (0, 0.25, 0.5, 1, and 2%) and glucose (10% *w*/*v*) as the carbon source. The formulated tea medium was sterilized by autoclaving at 121 °C for 60 min and then cooled to room temperature. The cooled medium was aliquoted (50 mL per vessel) and inoculated with 4% (*v*/*v*) activated starter cultures consisting of yeast and AAB at a 1:1 ratio. The fermentations were carried out statically at 28 °C for 9 days.

### 2.3. HPLC Profiling of Kombucha Metabolites

The HPLC procedure was adapted from Miranda et al. [27]. Prior to analysis, the samples were filtered through a 0.22 μm polyvinylidene difluoride (PVDF) membrane and transferred to HPLC vials. Chromatography was performed on a Hitachi HPLC system (Hitachi High-Tech, Tokyo, Japan) equipped with a UV detector (model L-2400). Separation was performed on a reversed-phase C18 column (Phenomenex, Torrance, CA, USA, 5 μm, 150 mm × 4.6 mm) maintained at 30 °C. The mobile phase consisted of (A) 0.1% (*v*/*v*) H_3_PO_4_ in water and (B) methanol, delivered at a flow rate of 0.5 mL/min using the following gradient program: 0 min, 80% A; 6 min, 60% A; 9 min, 80% A; and then followed by re-equilibration for 1 min. The injection volume was 10 μL. UV detection was set at 210 nm for organic acids and 273 nm for caffeine. External standards (glucuronic acid, ascorbic acid, gallic acid, acetic acid, succinic acid, and caffeine; Jing Ming Biomedical Technology Co., Ltd., Taipei, Taiwan, with stated purities ≥98% according to supplier specifications) were used for peak identification and quantification.

### 2.4. Antioxidant Capacity

DPPH scavenging activity was determined using a modified method of Jayabalan et al. [28]. Briefly, the samples (20 μL) were mixed with 0.1 mM DPPH in methanol (180 μL) in a 96-well microplate and incubated for 20 min at room temperature in the dark. The absorbance was measured at 517 nm using a microplate reader (SPECTROstar Nano, BMG LABTECH, Ortenberg, Germany). Each sample was assayed in 3 technical replicates for each biological replicate. DPPH scavenging activity was calculated as:

(1) $$DPPH\;scavenging\;activity\;(\%)=\left[1-\right.\left.\frac{A_{s}-A_{b}}{A_{c}}\right]\times 100$$

where A_s_ is the absorbance of sample plus DPPH solution; A_b_ is the sample blank (sample plus MeOH); and A_c_ is the control (MeOH plus DPPH solution). Trolox standards (7.5–300 μM) were freshly prepared in methanol and used to generate a calibration curve (R^2^ ≥ 99.6) for reporting results as Trolox equivalent antioxidant capacity (TEAC).

### 2.5. Polyphenolics Analysis

The total phenolic content (TPC) was determined using the Folin–Ciocalteu assay with minor modifications [29]. Aliquots of the samples (50 μL) were dispensed into a 96-well microplate and mixed with Folin–Ciocalteu reagent (50 μL, 0.4 N). After 5 min, sodium carbonate solution (100 μL, 6% *w*/*v*) was added, and the reaction was allowed to proceed for 30 min at room temperature in the dark. The absorbance was recorded at 765 nm using a microplate reader. TPC was quantified against a gallic acid calibration curve and expressed as mg gallic acid equivalents per milliliter (mg GAE/mL).

### 2.6. Bacterial Cellulose Production, Water Content and Swelling Ratio

Harvested KBC pellicles were purified by alkaline treatment using 0.1% (*w*/*v*) NaOH, followed by thorough rinsing under running tap water and an additional overnight soaking in water to remove residual alkali and soluble impurities. The purified BC was subsequently freeze-dried to a constant mass.

BC yield was expressed on a dry-weight basis and normalized to a culture volume (g/L). Briefly, the purified BC pellicles were weighed immediately after purification to obtain the wet mass (W_t_) and then freeze-dried to a constant weight to obtain the dry mass (W_d_). BC yield was calculated as W_d_/V, where V is the fermentation volume (L). Water content was calculated as:

(2) $$Water\;content\;(\%)=\frac{W_{t}-W_{d}}{W_{t}}\times 100$$

The swelling capacity was evaluated by re-immersing freeze-dried BC in distilled water until equilibrium. After rehydration, the samples were gently blotted to remove surface water and weighed to obtain the swollen mass (W_s_). After recording W_s_, the samples were freeze-dried again to a constant mass and reweighed to obtain the re-dried mass (W_d,re_). The swelling ratio was calculated as:

(3) $$Swelling\;ratio\;(\%)=\frac{W_{s}-W_{d,re}}{W_{s}}\times 100$$

### 2.7. Morphological and Physicochemical Characterization

All characterization analyses were performed using freeze-dried KBC samples.

#### 2.7.1. FTIR Analysis

Fourier-transform infrared (FTIR) spectra of KBC were acquired using a Spectrum 100 FTIR spectrometer (PerkinElmer, Wellesley, MA, USA) to identify the characteristic functional groups. Spectra were collected over 600–4000 cm^−1^ with 30 accumulated scans.

#### 2.7.2. SEM Analysis

The microstructure of KBC was examined by scanning electron microscopy (SEM; Hitachi S-4800, Tokyo, Japan). The samples were sputter-coated with gold prior to imaging. Micrographs were obtained at an accelerating voltage of 15 kV and a magnification of 1.0 × 10^4^.

#### 2.7.3. TGA Analysis

Thermogravimetric analysis (TGA; Q50, TA Instruments, New Castle, DE, USA) was conducted to evaluate the thermal stability of KBC. The samples were ground into a fine powder using a planetary ball mill (Retsch Mixer Mill MM 400; Sunpro International Technology Inc., Taipei, Taiwan). Approximately 5–10 mg of powder was placed in a platinum crucible and heated from 90 to 800 °C at 10 °C·min^−1^ under a nitrogen purge (40 mL·min^−1^) to minimize oxidative effects. The resulting thermogravimetric (TG) and derivative thermogravimetric (DTG) curves were analyzed to characterize the thermal degradation behavior.

### 2.8. Statistical Analysis

All experiments were performed in triplicate (at minimum), and the data are presented as mean ± standard deviation (SD). Statistical analyses were conducted using GraphPad Prism 10 (GraphPad Software, San Diego, CA, USA). The differences among groups were evaluated by one-way analysis of variance (ANOVA) followed by Tukey’s honestly significant difference (HSD) post hoc test, with statistical significance set at *p* < 0.05. Notably, several metabolites displayed non-linear responses to the male fig dosage, indicating a formulation-dependent modulation rather than a strictly monotonic trend.

## 3. Results

### 3.1. Physicochemical Analysis of FK

#### 3.1.1. Modulation of Organic Acids and Caffeine

**Figure 1:**
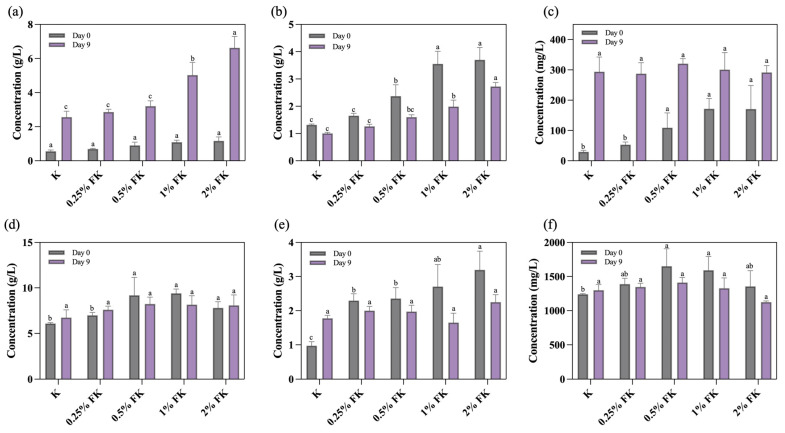
Organic acid and caffeine concentrations in FK at day 0 and day 9 of fermentation: (**a**) glucuronic acid; (**b**) vitamin C; (**c**) gallic acid; (**d**) acetic acid; (**e**) succinic acid; and (**f**) caffeine. Data are expressed as mean ± SD (n = 3). Within each group (same-colored bars), different superscript letters indicate significant differences (*p* < 0.05; one-way ANOVA with Tukey’s post hoc test).

As shown in Figure 1, fermentation for 9 days markedly altered the metabolite profile of FK. Glucuronic acid (Figure 1a) increased significantly in all groups and displayed a dose-associated trend with male fig supplementation, reaching the highest level in 2% FK (6.63 g/L) and the lowest in the control kombucha without the male fig (2.56 g/L) at day 9. Vitamin C (Figure 1b) decreased from day 0 to day 9 in every group; however, 2% FK consistently exhibited the highest vitamin C content at both time points. Gallic acid (Figure 1c) increased significantly after fermentation, peaking in 0.5% FK at day 9 (320.75 mg/L). Acetic acid (Figure 1d) showed concentration-dependent differences across the groups, with the highest value at day 9 observed in 0.5% FK (8.24 g/L). Succinic acid (Figure 1e) also varied by formulation, with the highest concentration at day 9 detected in 0.25% FK (2.60 g/L). Caffeine (Figure 1f) decreased over fermentation in all male fig-containing groups, whereas the control group (0% FK) showed an increase from day 0 to day 9.

#### 3.1.2. Optical Appearance and Color Difference

Table 1 summarizes CIELab color parameters of FK relative to the control kombucha (K) at day 0 and day 9. Across all formulations, L* increased from day 0 to day 9, indicating an overall increase in lightness during fermentation. The control K showed the highest lightness at day 9 (L* = 34.79), whereas all FK samples remained lower (L* ≈ 27.77–28.28), indicating that the male fig supplementation yielded darker beverages than the control at the end of fermentation.

#### 3.1.3. Antioxidant Capacity and TPC

Table 2 compares the antioxidant capacity of FK formulations on the final day of fermentation using DPPH (mM TEAC) and total phenolic contents (TPC, mg GAE/g). Numerically, all FK groups showed higher mean DPPH values (9.25–10.79 mM TEAC) than the control kombucha (K; 8.52 ± 2.20 mM TEAC). TPC also increased from 0.69 ± 0.46 mg GAE/mL (K) to 1.10–1.96 mg GAE/mL in FK samples, with the highest mean TPC observed in 1% FK (1.96 ± 0.87 mg GAE/mL); however, the differences were not significant (*p* ≥ 0.05).

### 3.2. Material Characteristics of FK-Derived BC

#### 3.2.1. BC Yield, Water Content, and Swelling Ratio

**Figure 2:**
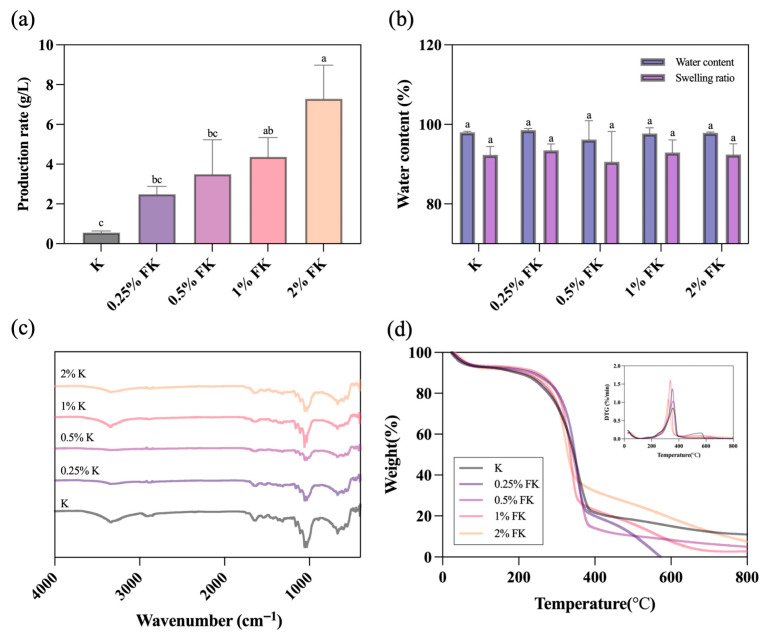
(**a**) BC yield expressed as dry mass normalized to culture volume (g/L). Data are presented as mean ± SD (n = 3). (**b**) Water content and swelling ratio of BC produced from FK fermentations. Data are presented as mean ± SD (n = 3). Within each group (same-colored bars), different superscript letters indicate significant differences (*p* < 0.05; one-way ANOVA with Tukey’s post hoc test). (**c**) FTIR spectra of BC, illustrating functional groups and substrate-dependent chemical features associated with FK fermentation. (**d**) Thermogravimetric (TG) and derivative thermogravimetric (DTG) curves of BC measured under nitrogen: TG was recorded from room temperature to 800 °C and DTG was derived from TG data acquired from room temperature to 800 °C, both at a heating rate of 10 °C·min^−1^. The temperature of maximum mass-loss rate (T_max) was obtained from the principal DTG peak.

As shown in Figure 2a, the male fig supplementation substantially increased the BC yield. The control kombucha (K) produced the lowest yield (0.56 ± 0.08 g/L), whereas yields increased to 2.49 ± 0.40 g/L (0.25% FK), 3.50 ± 1.73 g/L (0.5% FK), 4.36 ± 0.97 g/L (1% FK), and reached the highest value in 2% FK (7.28 ± 1.70 g/L). The hydration-related properties of KBC are presented in Figure 2b. Water content values were high across all samples (approximately 96–99%), while the swelling ratios after re-immersion were approximately 90–94%. No significant differences were detected among groups for either the water content or swelling ratio.

#### 3.2.2. FTIR

FTIR spectra of KBC obtained from the control kombucha and FK groups after 9 days (Figure 2c) exhibited the characteristic bands of cellulose. All samples showed a broad O–H stretching band at ~3350 cm^−1^ and a C–H stretching band at ~2920 cm^−1^, together with prominent carbohydrate fingerprint peaks in the 1200–900 cm^−1^ region (notably ~1110 and ~1030 cm^−1^) corresponding to C–O–C/C–O vibrations of the β-1,4-linked glucan backbone [30,31]. A band near ~1635 cm^−1^ was also observed across the samples [31,32]. The overall similarity of spectra indicates that male fig supplementation did not alter the fundamental cellulose chemical structure of KBC. Minor intensity/shape differences in the O–H stretching region and around ~1635 cm^−1^ likely reflect formulation-dependent changes in hydrogen-bonding environments and the association of bound/adsorbed water within the nanofibrillar network, rather than the formation of new covalent functionalities [31,32].

#### 3.2.3. TGA

As shown in the TG and DTG profiles (Figure 2d), all KBC samples exhibited the characteristic multistage thermal behavior of cellulose-based materials, comprising a low-temperature mass-loss region (below ~150 °C), a dominant main degradation stage at mid temperatures, and a final high-temperature residue. Quantitatively, the male fig supplementation increased early-stage thermal stability, reflected by higher T_5_% values relative to the control. The control K showed T_5_% = 50.36 °C, whereas FK groups showed elevated T_5_% values of 60.53 °C (0.25% FK); 57.72 °C (0.5% FK); 59.35 °C (1% FK); and 62.66 °C (2% FK). Likewise, T_10_% increased from 195.61 °C in K to 235.95 °C (0.25% FK); 209.35 °C (0.5% FK); 247.80 °C (1% FK); and 213.60 °C (2% FK). The maximum mass-loss rate temperature (T_max) was determined from the principal DTG peak and showed formulation dependence. The control exhibited T_max = 355.68 °C, while the FK samples exhibited 353.43 °C (0.25% FK) and 358.54 °C (0.5% FK), followed by a lower T_max in the higher male fig formulations (337.68 °C for 1% FK and 322.47 °C for 2% FK). The residual mass at high temperature also differed among groups, where at 600 °C, K retained 14.99%, while FK samples retained 8.31% (0.5% FK), 7.68% (1% FK), and 19.02% (2% FK). At the terminal temperature in the TG dataset (~800 °C), the residues were 10.86% (K); 4.83% (0.5% FK); 2.76% (1% FK); and 7.39% (2% FK). For 0.25% FK, the TG output approached ~0% and slightly below zero at high temperature, consistent with a baseline/normalization drift rather than a physically meaningful negative residue; therefore, its high-temperature residue was treated as approximately zero in interpretation.

#### 3.2.4. Morphological Analysis

**Figure 3:**
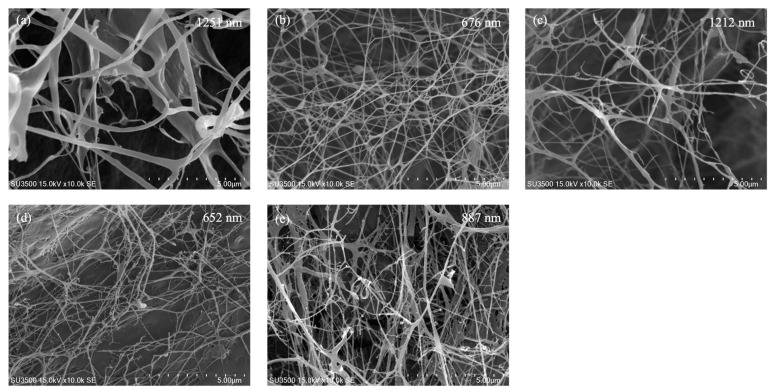
SEM images of BC collected from FK. Values are presented as means (n = 50). (**a**) Kombucha, (**b**) 0.25% FK; (**c**) 0.5% FK; (**d**) 1% FK; and (**e**) 2% FK.

SEM images (Figure 3) revealed that all KBC samples formed an interconnected, web-like fibrillar network typical of bacterial cellulose. Clear formulation-dependent differences were observed in fiber thickness and bundling. The control KBC (Figure 3a) exhibited comparatively coarser, ribbon-like bundles, with a representative fiber diameter of 1251 nm. Upon male fig addition, the network morphology shifted toward finer fibrils in some formulations; for example, 0.25% FK (Figure 3b) and 1% FK (Figure 3d) showed the thinnest fibers (676 nm and 652 nm, respectively) and a more densely entangled nanofibrillar mesh. In contrast, 0.5% FK (Figure 3c) displayed a thicker, more aggregated morphology with a larger fiber diameter (1212 nm), resembling a partial re-bundling of fibrils. The 2% FK (Figure 3e) sample showed an intermediate morphology (887 nm), maintaining a continuous nanofiber network while exhibiting moderate bundling compared with the finest fiber groups.

## 4. Discussion

Fermentation significantly modulated the metabolite profile of FK, and several responses were dose- or formulation-dependent. The increase in glucuronic acid across all groups, with the highest level in 2% FK, is consistent with the ability of AAB to shape organic-acid outputs in kombucha systems under defined process conditions [33]. Glucuronic acid increased after fermentation and reached 6.63 g/L in 2% FK, indicating that the male fig supplementation modulated the organic-acid composition under the present defined co-culture conditions. This level is higher than values reported in a prior study of traditional kombucha fermentation (maximum ~2.3 g/L), suggesting that the current formulation can substantially elevate glucuronic acid relative to some previous reports [34]. Vitamin C declined during fermentation, consistent with the chemical lability of ascorbic acid under conditions influenced by oxygen, pH, and temperature [35]. The higher vitamin C values retained at higher male fig dosages are compatible with matrix-dependent stabilization effects reported for low methoxyl pectin systems [36]. The post-fermentation rise in gallic acid aligns with kombucha biotransformation processes that release or transform phenolics during fermentation [21,37]. Differences in acetic and succinic acids among formulations likely reflect substrate-driven shifts in microbial metabolism and yeast–AAB interactions that govern organic-acid fluxes in kombucha [33]. Succinic acid exhibited formulation-dependent non-linear changes, consistent with the known sensitivity of succinate formation to fermentation conditions and microbial metabolism [38]. Caffeine decreased in male fig-containing groups but increased in the control; therefore, given that caffeine degradation is limited to specific microorganisms or pathways, the observed divergence may reflect matrix-dependent partitioning or limited transformation under the present co-culture conditions [39].

Color development was also strongly formulation dependent. CIELab trends showed that male fig supplementation darkened FK at day 9 and produced a concentration-dependent increase in ΔE*, indicating tunable appearance changes [40,41]. The pronounced increase in b* (yellowness) in the control after fermentation is consistent with tea polyphenol oxidation/polymerization and pigment evolution that can shift color toward yellow–brown hues [42,43]. In contrast, lower b* values in FK suggest that male fig components may modulate optical properties and/or phenolic reaction pathways, potentially through polysaccharide–polyphenol interactions that influence colloidal structure and chromophore development [44,45]. Because appearance is a key driver of consumer perception and product consistency, male fig dosages provide a practical lever for tuning fermentation color outcomes [46]. Future work linking instrumental color metrics to chemical chromophores (e.g., polymerized phenolics and browning indices) would clarify the dominant chemical drivers of FK color divergence [47,48].

Although DPPH and TPC values increased numerically in FK, the lack of statistical significance suggests that biological variability and matrix effects limited resolution under the current replication depth. This direction is consistent with reports that kombucha fermentation and substrate supplementation can elevate antioxidant readouts via phenolic release and microbial transformation [8,49,50,51]. Interpretation of TPC should remain cautious because Folin–Ciocalteu captures the overall reducing capacity rather than phenolics alone and is sensitive to assay conditions and interfering reductants [52,53]. Likewise, DPPH outcomes can vary with solvent compatibility, turbidity, and colored matrices, which may contribute to variability in fermented beverages [54]. Expanded replication, complementary antioxidant assays, and targeted phenolic profiling would strengthen inference regarding optimal male fig dosage [49].

From a materials perspective, the male fig supplementation substantially increased KBC yield in a dose-dependent manner, rising from 0.56 g/L in the control to 7.28 g/L in 2% FK. This magnitude of improvement is comparable with prior kombucha-based BC studies showing that the fermentation matrix strongly governs productivity. For example, kombucha fermentation on different herbal infusions (e.g., black/green tea, yerba mate, lavender, oregano, fennel) has been reported to alter BC film yield and process productivity while maintaining the cellulose-dominant chemical signature [26]. Likewise, kombucha-derived BC production from waste-derived substrates (e.g., whey-, apple juice-, and brewer’s spent grain–based media) has achieved higher yields (reported up to ~12–13 g/L) while preserving broadly similar BC physicochemical characteristics (FTIR/SEM/TGA), supporting the concept that nutrient-rich matrices can enhance BC output without necessarily disrupting the cellulose framework [55]. Importantly, despite the yield increase in this study, KBC hydration-related properties remained consistently high across formulations (water content ~96–99% and swelling ~90–94%), indicating that male fig supplementation enhanced productivity without a measurable loss of bulk water-holding or rehydration performance. This is consistent with prior kombucha–BC reports, in which matrix changes primarily affected productivity and microstructural features, whereas the cellulose backbone and key functional attributes were retained [26,56,57]. FTIR further supported the preservation of the cellulose backbone, with minor differences plausibly attributable to hydrogen-bonding microenvironments and bound-water association rather than formation of new covalent functionalities [30,31,32]. SEM revealed formulation-dependent fibril diameters and bundling, consistent with reports that BC network architecture responds to carbon sources and co-substrates during biosynthesis [45,58,59,60,61]. Finally, TGA indicated higher early-stage thermal stability (higher T_5_% and T_10_%) with dose-dependent shifts in the main degradation event, which can arise from altered microstructure and the presence of thermolabile non-cellulosic fractions associated with plant substrates [62]. Overall, these findings support male jelly fig syconia as a viable substrate for valorization into FK while simultaneously improving KBC productivity and maintaining desirable hydration-related material characteristics. Fermentation kinetics (pH, sugars, ethanol) and microbial counts (CFU) were not measured in this study, which limits mechanistic interpretation of the observed metabolite shifts. Thus, we focus on end-point compositional and KBC property outcomes, while time-resolved kinetic and CFU profiling will be addressed in future work.

## 5. Conclusions

This study demonstrates that male jelly fig syconia can be valorized as a fermentation substrate to produce FK using a defined starter culture. Male fig supplementation reshaped the chemical profile after 9 days of static fermentation, with glucuronic acid showing a dose-associated increase and vitamin C, gallic acid, and major organic acids exhibiting formulation-dependent responses, while caffeine decreased in all male fig-containing groups relative to the control. FK also displayed concentration-dependent divergence in CIELab color parameters, indicating that male fig dosage can be used to modulate product appearance under the conditions tested. Although DPPH and TPC increased numerically, differences were not statistically significant under the current replication depth. Importantly, male fig supplementation substantially enhanced KBC productivity, increasing yield from 0.56 g/L in the control to 7.28 g/L in 2% FK, while maintaining high water content and swelling capacity. FTIR confirmed the retention of the cellulose backbone, SEM showed a continuous porous nanofibrillar network with formulation-dependent fiber diameters, and TGA indicated an improved early-stage thermal resistance with dose-dependent modulation of degradation behavior, collectively suggesting that male fig addition primarily promoted cellulose biosynthesis without compromising key material attributes. Future studies should integrate targeted metabolomics with basic microbial monitoring to clarify pathways underlying glucuronic acid formation and phenolic transformations, and should strengthen antioxidant evaluation through increased replication, complementary assays, and targeted phenolic profiling, while linking instrumental color changes to defined chromophores. Notably, the present work reports compositional and material–property outcomes only; therefore, functional relevance and consumer-facing performance remain to be validated. Translation toward practical implementation will require routine monitoring of fermentation kinetics (pH, residual sugars, and ethanol), verification of co-culture dynamics (e.g., microbial counts), and robust process control, including oxygen availability and static culture geometry that influence BC formation, as well as standardized raw material specifications to manage male fig variability. In addition, scalable purification and drying workflows, together with sensory and safety validation, will be essential to ensure batch-to-batch consistency of FK and KBC products.

## Figures and Tables

**Table 1:** CIELab color parameters (L*, a*, b*) and color differences (ΔE*) of FK relative to the control at day 0 and day 9 of fermentation.

	Day 0				Day 9			
	L*	a*	b*	ΔE*	L*	a*	b*	ΔE*
K	26.10	10.00	11.12	-	34.79	13.14	24.82	-
0.25% FK	23.25	12.22	12.08	3.74	28.28	10.59	14.71	12.29
0.5% FK	22.98	13.14	10.89	4.43	28.28	10.62	12.54	14.13
1% FK	24.55	14.51	10.50	4.81	27.77	11.09	10.46	16.11
2% FK	25.94	14.37	9.42	4.69	27.96	12.35	8.39	17.81

K denotes kombucha; FK denotes Fig-altered kombucha.

**Table 2:** Comparison of the antioxidant capacity between FK samples on the final day of fermentation.

	DPPH (mM TEAC)	TPC (mg GAE/mL)
K	8.52 ± 2.20 ^a^	0.69 ± 0.46 ^a^
0.25% FK	10.32 ± 3.84 ^a^	1.10 ± 0.56 ^a^
0.5% FK	9.25 ± 1.00 ^a^	1.21 ± 0.47 ^a^
1% FK	10.79 ± 1.71 ^a^	1.96 ± 0.87 ^a^
2% FK	10.79 ± 1.86 ^a^	1.63 ± 0.32 ^a^

K denotes kombucha; FK denotes Fig-altered kombucha. Values are expressed as mean ± SD (n = 3). Superscript letter a indicate significant differences among groups (*p* < 0.05).

## Data Availability

The data presented in this study are available on request from the corresponding authors. The data are not publicly available as they form part of an ongoing research project.
